# Evaluation of knowledge about antibiotics and engagement with a research experience on antimicrobial resistance between pre-university and university students for five school years (2017–2021)

**DOI:** 10.3389/fmicb.2022.959187

**Published:** 2022-08-10

**Authors:** Antonio Tarín-Pelló, Beatriz Suay-García, Elisa Marco-Crespo, Carolina Galiana-Roselló, Jose I. Bueso-Bordils, María-Teresa Pérez-Gracia

**Affiliations:** ^1^Área de Microbiología, Departamento de Farmacia, Universidad Cardenal Herrera-CEU, CEU Universities, Alfara del Patriarca, Spain; ^2^ESI International Chair@CEU-UCH, Departamento de Matemáticas, Física y Ciencias Tecnológicas, Universidad Cardenal Herrera-CEU, Alfara del Patriarca, Spain; ^3^Departamento de Comunicación e Información Periodística, Universidad Cardenal Herrera-CEU, Alfara del Patriarca, Spain

**Keywords:** antimicrobial resistance, education, antibiotics, AMR awareness, rational use, citizen science, antimicrobial stewardship

## Abstract

Antimicrobial resistance (AMR) remains a serious global health problem. Spain is the fifth country in Europe with the highest consumption of antibiotics, due in part to ignorance of the good use of these drugs and the problem of AMR. To avoid a post-antibiotic era, adequate training on this problem is key to create social awareness. This study aimed to evaluate the impact that the SWICEU project, an academic program about antibiotic discovery, has had on the knowledge of AMR and rational use of antimicrobials in pre-university students from seven schools in the province of Valencia during five academic years (2017–2021), as well as to evaluate the level of satisfaction of university and pre-university students who have participated in the project. For this study, a survey was carried out with multiple-choice questions with a single correct answer to evaluate the knowledge acquired by pre-university students before and after the project. A satisfaction survey was also designed with a Likert scale from the lowest to the highest level of satisfaction for the two groups of students after the project. Data on knowledge surveys indicated an increase in the mean number of correct answers after the sessions. In satisfaction surveys, we highlighted the issue that referred to the project’s recommendation. The data obtained confirm this project as a valuable activity, as it allows learning about AMR and the rational use of antibiotics in a pleasing and attractive way for young pre-university and university students.

## Introduction

Since the discovery and application of the molecule sulfonamidocrisoidin (better known under the name of Prontosil^®^) and the discovery and development of penicillin during the 30s and 40s, humanity has lived a golden age regarding the treatment of bacterial infections (Dodds, 2017). The opportunity to combat these types of diseases has saved thousands of lives throughout history, thanks to the continuous research and development of new antimicrobial drugs. However, Alexander Fleming already warned in his speech after obtaining the Nobel Prize about the danger posed by the appearance of bacteria resistant to antibiotics due to the misuse of these molecules, making clear the importance of training and knowledge about the rational use of these drugs not only to health professionals, but also to the rest of the population (Fleming, 1945).

Currently, antibiotic resistance has become a serious health problem worldwide (León-Buitimea et al., 2020), listed by the World Health Organization (WHO) as one of the top 10 public health threats facing humanity (World Health Organization [WHO], 2019). According to the European Centre for Disease Prevention and Control (ECDC), Spain is the fifth country in Europe with the highest consumption of antibiotics (European Centre for Disease Prevention and Control [ECDC], European Food Safety Authority [EFSA], and European Medicines Agency [EMA], 2021), due to ignorance of the rational use of these medicines (European Commission [EC], 2018). In this last document, it was observed that 48% of the European population surveyed thought that antibiotics act against viruses, and 19% claimed to take antibiotics to treat the flu and cold (European Commission [EC], 2018). In addition, 66% of the people surveyed said they did not remember receiving information about not taking antibiotics in the case of suffering from one of these two diseases. The lack of knowledge about the rational use of these drugs contributes to the increase in the frequency of the appearance of antimicrobial resistance (AMR), non-compliance with the optional prescription, loss of adherence to treatment, self-medication, and the lack of hygienic-sanitary measures, such as hand washing or vaccines, key factors for adequate prophylaxis and, consequently, for a decrease in antimicrobial consumption (Dyar et al., 2016).

According to the WHO, if this situation does not improve by 2050, bacterial infections will be the leading cause of mortality worldwide, attributing a number of deaths close to 10 million people each year; outnumbering traffic accidents, cancer, and diabetes. This figure is equivalent to the population of countries, such as Sweden, the Czech Republic, Greece, or Portugal (Eurostat, 2016). To avoid this alarming future, the WHO proposed prevention and control measures for the spread of bacterial resistance (World Health Organization [WHO], 2018). Regarding the social and professional field, continuous training is proposed through different methods, such as the use of the media, conferences of specialists in the subject, and Antimicrobial Optimization Programs (Dyar et al., 2016). This training is key in raising awareness of the global danger of antibiotic resistance, as well as the benefits of adequate antibiotic use (Global Leaders Group on Antimicrobial Resistance, 2021).

The international Small World Initiative (SWI) program appeared as an awareness proposal aimed at young university students. Dr. Jo Handelsman, a professor in the Department of Molecular, Cellular and Developmental Biology at Yale University, was aware that the number of students entering STEM (Science, Technology, Engineering, and Mathematics) majors was declining every year (SmallWorldInitiative^®^ CrowdsourcingAntibioticDiscovery™, 2015). With the aim of increasing motivation toward choosing a degree in Experimental Sciences and contributing to the search for new antibiotics, in 2012, Dr. Handelsman initiated the SWI program. This initiative focuses on a discovery-based basic microbiology course; in which students conduct fieldwork and research in the laboratory on soil samples in search of new antibiotics. In 2016, the Complutense University of Madrid introduced this program in Spain, integrating two educational levels, university and pre-university students, through a service-learning strategy (Universidad Complutense de Madrid, 2016). Through this strategy, a social contribution is obtained that is summarized in two aspects: on one hand, future university students are involved in a project that encourages scientific interest and motivates training aimed at Experimental and Health Sciences. On the other hand, it provides knowledge and social awareness about the problem of AMR and the rational use of antibiotics. During the 2017–2018 academic year, the CEU Cardenal Herrera University implemented this project calling it SWICEU, which was taken to seven schools in the province of Valencia. As a result of this initiative, the MicroMundo project was born, sponsored by the Spanish Society of Microbiology (SEM), in which more than 30 universities in Spain and Portugal are involved. In 2018, the SWI program was refounded as Tiny Earth (2018), in which more than 200 universities worldwide are currently involved, including our CEU Cardenal Herrera University (SWICEU UCH, 2017-2022).

Due to the scope and importance at an international level of the problem of AMR, the aim of this study was to evaluate the impact that the SWICEU project has had on the knowledge about antibiotics and AMR in pre-university students of seven schools in the province of Valencia for five academic years (2017–2021). In addition, the degree of satisfaction of the levels of participation in the project corresponding to pre-university and university students was also evaluated.

## Materials and methods

The SWICEU project consists of five practical laboratory sessions, where pre-university students look for microorganisms that produce antimicrobial molecules from soil samples, in addition to being aware of the problem of antibiotic resistance and the rational use of these drugs. During the five academic years (2017–2021), the sessions were taught by several teams formed by three and five undergraduate students led by a university professor. The role of our university (undergraduate) students was to act as teaching assistants in each school where they taught classes. To do this, they prepared their presentations in a way that they were entertaining and interesting. In addition, they prepared all the laboratory material and culture media necessary for each practice session.

For the evaluation of the knowledge acquired during the project by the pre-university students, a survey was designed consisting of 12 multiple-choice questions with a single valid answer (Supplementary Figure 1). This survey was written in Spanish and English and was conducted both at the beginning and the end of the project. In this way, it was possible to observe the changes in the surveys and evaluate the level of knowledge acquired on the appropriate use of antibiotics and the level of perception about the problem of AMR after the educational component of the project. This survey was prepared based on questions asked by students and university professors who participated in the SWICEU project. It was decided to carry out the surveys anonymously so as not to exert added pressure on the students and to preserve the relaxed, although the rigorous, character of the activities carried out.

For the evaluation of the satisfaction of the pre-university students after the project, a survey was designed that consisted of 11 questions on a Likert scale. Every question had five boxes to answer with an X, the first box being the value with the most negative aspect (value 1) and the fifth box being the most positive aspect (value 5) (Supplementary Figure 2). In this way, the degree of greatest satisfaction would correspond to 55 points. With this structure, issues such as scientific interest, the perception of learning about antibiotic resistance, among other issues, were evaluated. Three opinion spaces were also added where the students wrote the most positive, negative, and improved aspects of the project. With the same mechanics, a survey was designed for university students. In this case, there were 18 questions related to the same issues named, in addition to learning about good laboratory practices and the ability to transmit the project to high school students (Supplementary Figure 3). In this case, the highest degree of satisfaction would correspond to 90 points.

### Personal data protection

All the people evaluated in the SWICEU project were previously informed about their evaluation processes, the use of their personal data in written and audiovisual content, as well as their rights as individuals. All participants in the project gave their consent.

### Statistical analysis

Statistical parameters (such as mean, standard deviation, and variances) were calculated using the Microsoft^®^ Excel^®^ program for Microsoft 365 MSO version 2204. We used the Fisher-Snedecor test (*F*-test) to analyze the equality of variances and the Student’s *t*-test to determine statistical significance, both tests were performed at the 95% confidence interval (CI).

## Results

### Number of students surveyed

The number of pre-university students of 4th ESO (10th grade in the United States) and 1st Baccalaureate (11st grade in the United States) who participated in the project was 470, belonging to seven schools in the Valencian Community (Spain). There were 140 university students, belonging to various grades of the Health Sciences degrees (Pharmacy, Medicine, Veterinary, Dentistry, and Nursing), Communication and Design Engineering. The study was conducted over five academic years, from 2017 to 2021. The distribution of pre-university and university students by the course was as follows: 90 and 25 in 2017–2018, 132 and 40 in 2018–2019, 100 and 30 in 2019–2020, 56 and 20 in 2020–2021, and 92 and 25 in 2021–2022, respectively. It is important to note that due to the pandemic caused by SARS-CoV-2 and the protocols carried out in schools, the number of students was lower in the 2020–2021 academic year.

### Evaluation of the knowledge acquired during the project by pre-university students

**FIGURE 1:**
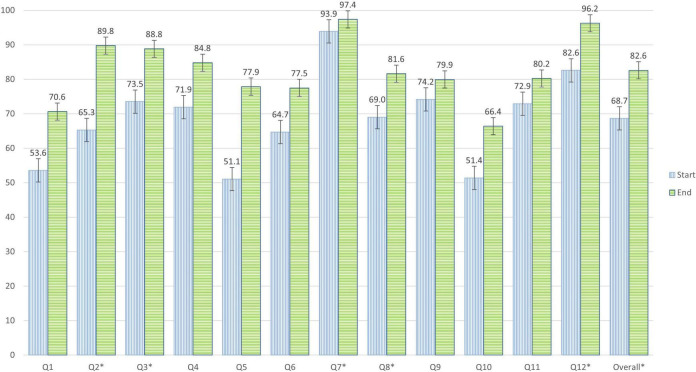
Percentages of correct answers to the 12 questions of the survey carried out by pre-university students at the beginning and end of the SWICEU project during the 2017–2021 academic years. “*”Marks the questions with statistically significant differences.

After analyzing the results of the survey, we observed statistically significant differences in the general average of the five academic years, with 68.7% (8.2/12) of successes at the beginning and 82.6% (9.9/12) at the end of the project (Figure 1). These data indicate that students correctly answered an average of 1.7 more questions after completing the practical sessions of the project. Differences in results before and after the project have been found to be statistically significant with a 95% CI for questions 2, 3, 7, 8, and 12 (Table 1). The rest of the questions asked to pre-university students (1, 4, 5, 6, 9, 10, and 11) did not obtain statistically significant differences.

**TABLE 1:** Statistical parameters at a 95% confidence interval of the comparison between results obtained at the start and at the end of the SWICEU project.

	Average	Standard deviation	Variance	Covariance	*F*-test	Variance equality	Weighted variance	*t*-test	*r*	*r* ^2^	*p*	Significant differences
Q1 start	53.563	14.853	176.482	–42.799	0.941	Equal	214.048	1.846	–0.250	0.063	0.102	No
Q1 end	70.642	14.405	207.493									
Q2 start	65.304	9.573	73.315	15.992	8.208	Unequal	51.405	5.391	0.625	0.391	0.003	Yes
Q2 end	89.748	3.342	11.166									
Q3 start	73.526	8.829	62.355	3.917	6.137	Equal	45.322	3.592	0.156	0.024	0.007	Yes
Q3 end	88.821	3.564	12.701									
Q4 start	71.911	12.388	122.773	65.245	2.627	Equal	105.945	1.979	0.861	0.742	0.083	No
Q4 end	84.792	7.644	58.423									
Q5 start	51.058	29.828	711.783	0.552	8.827	Unequal	495.262	1.904	0.002	5.31*10^–6^	0.115	No
Q5 end	77.860	10.040	100.795									
Q6 start	64.669	9.712	75.462	124.017	4.235	Equal	246.921	1.289	0.799	0.638	0.234	No
Q6 end	77.476	19.988	399.514									
Q7 start	93.875	2.176	3.787	0.101	6.263	Equal	2.745	3.327	0.067	0.004	0.010	Yes
Q7 end	97.361	0.869	0.756									
Q8 start	69.004	5.394	23.276	16.118	1.414	Equal	24.833	3.999	0.824	0.678	0.004	Yes
Q8 end	81.606	4.535	20.570									
Q9 start	74.152	6.163	30.386	19.685	1.475	Equal	31.868	1.621	0.787	0.619	0.144	No
Q9 end	79.939	5.075	25.754									
Q10 start	51.368	11.511	106.003	–13.627	1.132	Equal	141.282	1.998	–0.121	0.015	0.081	No
Q10 end	66.388	12.250	150.059									
Q11 start	72.919	11.168	99.778	–19.257	2.967	Equal	83.383	1.266	–0.332	0.110	0.241	No
Q11 end	80.229	6.484	42.043									
Q12 start	82.597	9.820	77.154	6.317	24.670	Unequal	50.176	3.045	0.407	0.165	0.038	Yes
Q12 end	96.241	1.977	3.909									
Overall start	68.662	4.138	13.699	0.376	3.292	Equal	11.162	6.592	0.050	0.002	1.71*10^–4^	Yes
Overall end	82.592	2.281	5.201									

When analyzing the results before and after the project, we highlight the following results in global data: In question 2, “Is a prescription necessary to buy antibiotics?,” 65.3% answered correctly at the beginning and 89.7% at the end of the practical sessions (Supplementary Figure 9). In question 3, “Which of the following drugs is an antibiotic?,” 73.5% of the students surveyed answered correctly at the beginning and 88.8% at the end of the project (Supplementary Figure 10). To question 7, “If we do not take antibiotics correctly:,” 93.9% answered correctly at the beginning and 97.4% at the end of the practical sessions (Supplementary Figure 11). The results of question 8, “What is antibiotic resistance?,” showed that 69% of the students surveyed answered correctly at the beginning of the project and 81.6% at the end (Supplementary Figure 12). Finally, in question 12, “According to the World Health Organization (WHO), in 2050 it is estimated that the first cause of death will be:” 82.6 and 96.2% of correct answers were observed at the beginning and end of the project, respectively (Supplementary Figure 13).

### Evaluation of the satisfaction of pre-university students after the completion of the project

**FIGURE 2:**
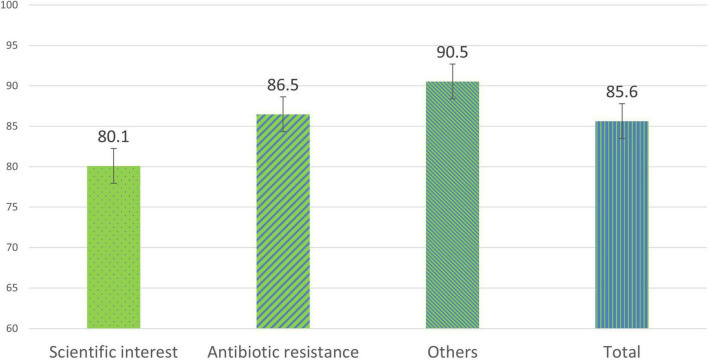
Percentages of the average satisfaction values of each general section in pre-university students at the end of the SWICEU project during the 2017–2021 academic years.

**FIGURE 3:**
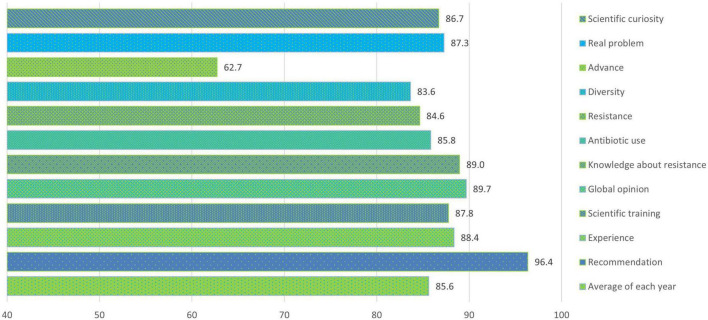
Percentages of the average values of satisfaction of each issue in pre-university students at the end of the SWICEU project during the 2017–2021 academic years.

The analyses carried out on the satisfaction surveys of pre-university students show an overall average satisfaction of 85.6% (47/55) at the end of the project (Figure 2). The section dedicated to the scientific interest transmitted presented a lower average value compared to the rest of the sections with 80.1%. The Other section, with questions of general valuation and recommendation, presented the highest value with 90.5%. As for the questions in each section, those related to whether the project contributes to an advance in science with an average value of 62.7% and whether they recommend other students to participate in this project with 96.4% stand out (Figure 3).

In the opinions of pre-university students, in the fields of the open response of the survey, issues such as the improvement of knowledge of good laboratory practices and the research work carried out in the sessions were named, in addition to the participation of the student in a real problem in an active way. These issues were valued as the best that the project has contributed during the five courses.

In the opinions of the pre-university students, in the open response section of the survey, topics such as the improvement of knowledge of good laboratory practices and the investigative work carried out in the sessions were named, in addition to the active participation of the students in a real problem. These topics were rated as the best that the project has provided during the five academic years. Factors such as learning the rational use of antibiotics and the problem of antibiotic resistance were also positively valued, in addition to increasing curiosity and interest in science in an amusing and enjoyable way for students. The positive attitude of the university students and their ability to solve the doubts of the pre-university students were appreciated. As for negative opinions, we found dissatisfaction in the students when it came to not finding antibiosis in their soil sample and the short duration of the practices, having an interest in discussing the results obtained during the sessions. In the same way, the aspects considered improvable by the pre-university students were: the duration of the project to carry out the sessions in a calmer and deeper way, with time to provide more information about what was done in the laboratory, the results obtained, and the contribution of these in the scientific community. The explanations of the practices to be carried out and the training on the problem of antibiotic resistance and the rational use of drugs were also considered improvable.

### Evaluation of the satisfaction of university students after the project

**FIGURE 4:**
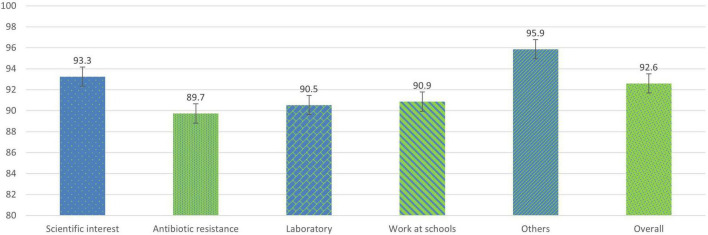
Percentages of the average satisfaction values of each general section in university students at the end of the SWICEU project during the 2017–2021 academic years.

**FIGURE 5:**
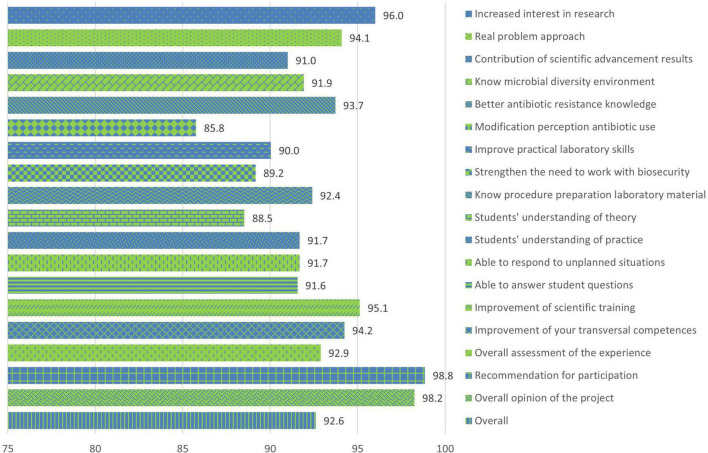
Percentages of the average satisfaction values of each issue in university students at the end of the SWICEU project during the 2017–2021 academic years.

After completing the project in the surveys carried out by university students, an overall average satisfaction of 92.6% (47/55) was observed at the end of the project (Figure 4). The general section dedicated to antibiotic resistance presented a slightly lower average value compared to the rest of the sections, with 89.7%. The general assessment section “Others” presented the highest value with 95.9%. Regarding the issues in each section, those related to the modification of the perception of the use of antibiotics with an average value of 85.8% and the recommendation to participate in the project with 98.8% satisfaction (Figure 5) stand out.

The opinions of the students were positive in terms of participating in an activity related to the service-learning strategy being them the “teachers” of the pre-university students, teaching them science and research in an entertaining and original way. They also expressed their satisfaction with the reinforcement of the knowledge of microbiology and pharmacology provided by the project, as well as the ability to demonstrate them in a practical way in the sessions. As for negative opinions and issues that can be improved, we observe similarities with the opinions of pre-university students, considering that the practical sessions should be more extensive to be able to interact more with the students and to be able to provide them with more information about the problem of antibiotic resistance, the rational use of these drugs, in addition to carrying out the sessions in a calmer way.

## Discussion

From the results of the surveys on the knowledge acquired during the project, an increase of 13.9% has been observed when comparing the number of questions answered correctly at the beginning and end of the sessions, we can affirm that there has been a significant overall improvement in the scientific knowledge of the pre-university students participating in the project. There are examples to support these results, such as the curriculum for antimicrobial stewardship education developed by the Infectious Diseases Society of America in 2021, which was well received by Infectious Diseases fellowship programs. Specifically, a pre- and post-implementation analysis of the curriculum revealed an increase in fellows’ confidence and a significant increase in knowledge of antimicrobial stewardship content areas compared to before the implementation of the curriculum (Spicer et al., 2021). In addition, the pre-university students themselves have reinforced this statement through positive satisfaction surveys, including learning about antibiotic resistance and their rational as the best of the project. However, the answers obtained to some of the questions deserve special attention.

Question 2, “Is a prescription necessary to buy antibiotics?,” underwent a very considerable change with an overall increase of 24.4% of correct answers given before and after the project (Supplementary Figure 9). These data correspond to the emphasis shown by students and university professors on the risks, both for the patient and for society, associated with the sale of antibiotics without a prescription (Guinovart et al., 2018). This attitude is reflected in the satisfaction surveys of pre-university students, which have included the good work and motivation with which the monitors have carried out the sessions as the best of the project. We highlight that, year after year, the percentage of correct answers at the end has remained with percentages between 85.7% (2017–2018) and 94.8% (2019–2020). We can affirm that the tendency to reduce the gap between the percentage of correct answers before and after the project is related to the fact that students had a better knowledge about this question at the beginning (48.9% of correct answers at the beginning of the 2017–2018 academic year, 65.2% in 2018–2019, 70.0% in 2019–2020, 69.6% in 2020–2021, and 72.8% in 2021–2022).

Question 3, “Which of the following drugs is an antibiotic?,” presented a notable difference compared to last year’s results (2021), with an overall increase of 15.3% in correct answers before and after the project (Supplementary Figure 10). These data are probably due to the training of university students in pharmacology, both for their undergraduate studies and for their training in the SWICEU project. It is important to note that in the SWICEU team there is a multidisciplinary group of students, where there are not only students with Health degrees, but students with Communication and Design Engineering degrees are also involved (Pérez-Gracia et al., 2018), who need additional training to better understand the differences between antibiotics and other types of medicines. This training provided by university professors for all university students could be the reason why during the five academic years the percentage of correct answers after completing the project has remained between 88.1% (2017–2018) and 93.1% (2018–2019) in the case of question 3, and for the same reason, question 7, “If we do not take antibiotics correctly:,” maintains average values between 95.9% (2019–2020) and 98.1% (2020–2021) at the end of the project (Supplementary Figure 11). This reason is supported by the data provided by the satisfaction surveys of the students themselves, where they express percentages of satisfaction between 93 and 95% on issues such as improving knowledge about antibiotic resistance and improving their transversal competences (Figure 5). The improvement in the training of university students has a direct impact on the learning of pre-university students. However, we found that the question regarding the modification of the perception of the use of antibiotics presents the lowest value in the satisfaction survey of university students, with 85.8%. This result, although positive, proves the importance of training for university students that allows them to perceive the relevance of the rational use of antibiotics. Only in this way can awareness be achieved in pre-university students about the good use of these medicines.

In question 8, “What is antibiotic resistance?,” there has been an overall increase of 12.8% in the correct answers from the beginning to the end of the practical sessions (Supplementary Figure 12). The positive results that we observe year after year may be due to the fact that this question directly covers the issue of antibiotic resistance, which is not only explained during the practices but is also recognized by the students in them when observing the inhibition halos of their samples. This recognition is reflected in the student satisfaction surveys, where the question related to the contribution of knowledge about the resistance of the project reaches 89.7% satisfaction. In addition, university students also recognize the best understanding of this problem with a percentage of satisfaction of 93.7% in the issue that refers to it. Also related to AMR, we have question 12, “According to the WHO, in 2050 it is estimated that the first cause of death will be,” which presents an increase of 13.6% in the number of correct answers at the end of the project (Supplementary Figure 13). This confirms that the level of awareness of the global health problem that is antibiotic resistance increases with the practical sessions and knowledge provided by the university to pre-university students. Finally, we must emphasize that the issues that have not obtained statistically significant results in our study, although they have presented positive results, confirm that it is possible to improve the sessions to reinforce the concepts of the project, such as the differences between bacteria and other microorganisms (question 1), bacterial infections (question 5), and the rational use of antibiotics (questions 4, 6, and 9–11).

It is important to note that, according to the information obtained from the surveys themselves, reasonably uniform results have been gathered throughout the academic years 2017–2021. It is also important to note that, due to the pandemic caused by the SARS-CoV-2 virus and the protocols carried out in schools, the number of students has been lower in the 2020–2021 academic year. This has allowed calmer sessions with more personalized attention, maintaining positive results. However, in the last year, we have returned to normal in the classrooms and we have reached the same population of pre-university students as in the 2017–2018 academic year, the first year when this project was carried out at our university, and positive results similar to the previous academic years (2017–2021) have been achieved.

From the satisfaction surveys of both pre-university and university students, we observed that, at a global level, the project is interesting, fun, and didactic. Issues such as increased interest in science and research, improved competencies related to good laboratory practices, and the opportunity to participate in a real research project, make more than 95% of participants recommend this project (Figures 2–5). However, it is important to improve several aspects for future editions. Pre-university students question the scientific progress that this project brings to society, with this question obtaining the lowest score in the survey (Figure 2). This result might be attributable to the fact that they considered that the experiment had failed if they did not observe antibiotic-producing colonies in their soil samples. The presence or not of antibiosis in the samples remains an important fact in the search for new antibiotics. The concept that a positive result (antibiosis) is just as relevant as a negative result (non-antibiosis) should be better explained in the coming courses.

As for university students, the change in the perception of pre-university students on the use of antibiotics seems to be improvable in the project sessions (Figure 5). This result may be due to the fact that the training on the rational use of these drugs, although explained throughout the sessions, is concentrated in the first and last sessions, as the first session focuses on introducing pre-university students to the context of the AMR problem, and the last session focuses on resolving any doubts they may have at the end of the project. It is likely that university students have observed that these concepts require more time and dedication in the sessions with the students. This observation corresponds to other questions and comments related to the theoretical explanation of pre-university students, where university and pre-university students exhibit the need to increase the time of the sessions to settle concepts and clarify doubts. The data obtained by the satisfaction surveys generally show positive results on the contribution of the project and help to improve the training of both university and pre-university students in future editions.

The fact of being able to carry out an experiment with a real first-hand purpose, without being a mere spectator, and being able to observe and interpret the results obtained next to professionals in the field, makes this project a comfortable, close, and didactic method that brings future generations closer to the scientific world, teaching important and current concepts, such as antibiotic resistance and the rational use of these drugs. The seriousness of the issue of AMR has been admitted by various governments and international organizations, developing different plans to combat this threat (Pérez-Gracia, 2021). Among the WHO proposals is the promotion of the rational use of antibiotics in all areas, including the social (World Health Organization [WHO], 2021). In the United Kingdom, the document “Addressing drug-resistant infections worldwide: final report and recommendations” presents various social actions to be carried out to curb the emergence of antibiotic resistance, such as conducting massive and global public awareness campaigns, improving hygiene, and reducing the unnecessary use of these drugs (O’Neill, 2016). The Global Leadership Group on AMR has also declared measures to be taken for all countries and leaders in different sectors, such as improvements in pharmacovigilance and monitoring of responsible and sustainable use of antimicrobials (Global Leaders Group on Antimicrobial Resistance, 2021). In Spain, the National Antibiotic Resistance Plan (PRAN) has structured various strategies to curb the problem of AMR, such as surveillance, control, and prevention of its appearance, as well as training and communication to the entire population about the problem and measures to be taken to avoid reaching a post-antibiotic era, where antibiotics are not able to treat infections (National Antibiotic Resistance Plan, 2021). These measures, strategies, and actions have been reflected in the results obtained year after year by the SWICEU team in this project, thanks to the pedagogical methodology of service-learning (Bueso-Bordils et al., 2020). This analysis demonstrates the urgent need for new and interesting educational and awareness-raising programs that integrate methods to optimize prevention and response to infectious diseases (McDonnell Norms Group, 2008; Hall et al., 2020; U.S. Antibiotic Awareness Week [USAAW], 2021). In this way, it is possible to understand the importance of these methods as sources of knowledge and awareness about the problem of AMR and the rational use of antimicrobials (Hayes, 2022).

## Conclusion

The overall experience of the SWICEU project remains positive year after year. Participation in this project demonstrates greater awareness of the appropriate use of antibiotics and information on AMRin pre-university students. In the same way, we can affirm that all the students participating in this project have improved their scientific knowledge. The greatest benefit provided by this project is reflected in the knowledge obtained by the pre-university students, who are able to transmit it to their social environment, further increasing awareness of this problem. Another benefit of the project is the satisfaction obtained by both pre-university and university students for participating in a project that works to improve society from different approaches to the problem of antibiotic resistance. We would also like to highlight that the SWICEU project shows the importance and need for new approaches to reach young people and the general public in the field of infectious diseases and the rational use of this drug. Thanks to these strategies, it is possible to educate the population in an original and attractive way, generating a society aware and involved in complex medical problems, such as AMR.

## Data availability statement

The original contributions presented in this study are included in the article/Supplementary material, further inquiries can be directed to the corresponding author.

## Author contributions

M-TP-G was responsible for the project design, conception, and management, integrity of the work, and overall supervision. AT-P, BS-G, CG-R, JIB-B, and M-TP-G performed the practicals. EM-C was responsible for the broadcasting of the project. AT-P wrote the first draft of the manuscript. BS-G, EM-C, CG-R, JIB-B, and M-TP-G wrote sections of the manuscript. All authors contributed to the interpretation of the data, manuscript revision, read, and approved the manuscript.
