# Supplementary material for: Evaluation of knowledge about antibiotics and engagement with a research experience on antimicrobial resistance between pre-university and university students for five school years (2017–2021)
Source: Front Microbiol. 2022 Aug 10;13:959187. doi: 10.3389/fmicb.2022.959187 (PMC9402252; doi:10.3389/fmicb.2022.959187)
Supplement: Supplementary file 1 [file Data_Sheet_1.docx]

# Supplementary Material


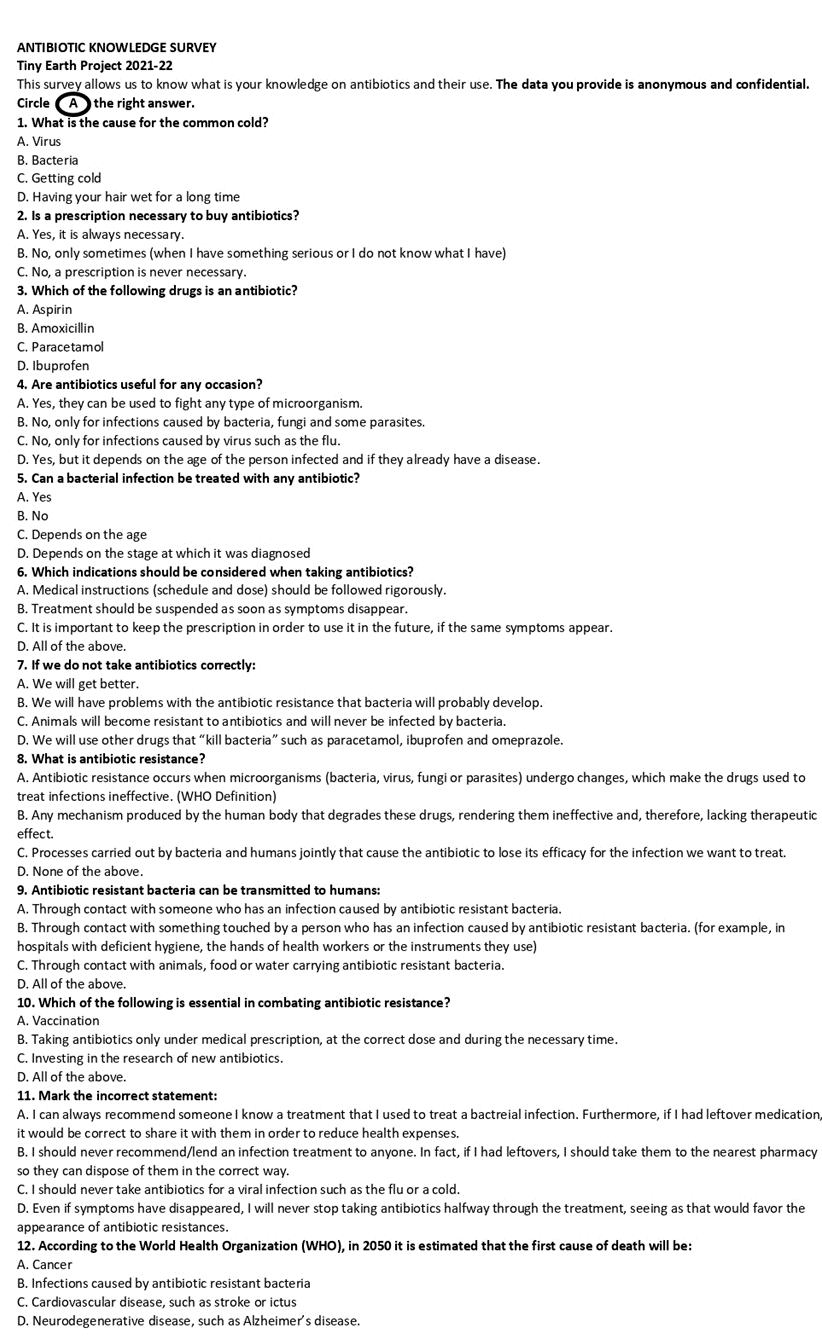


Supplementary Figure 1. Survey conducted at the beginning and end of the SWICEU project during the 5 academic years 2017-2021.


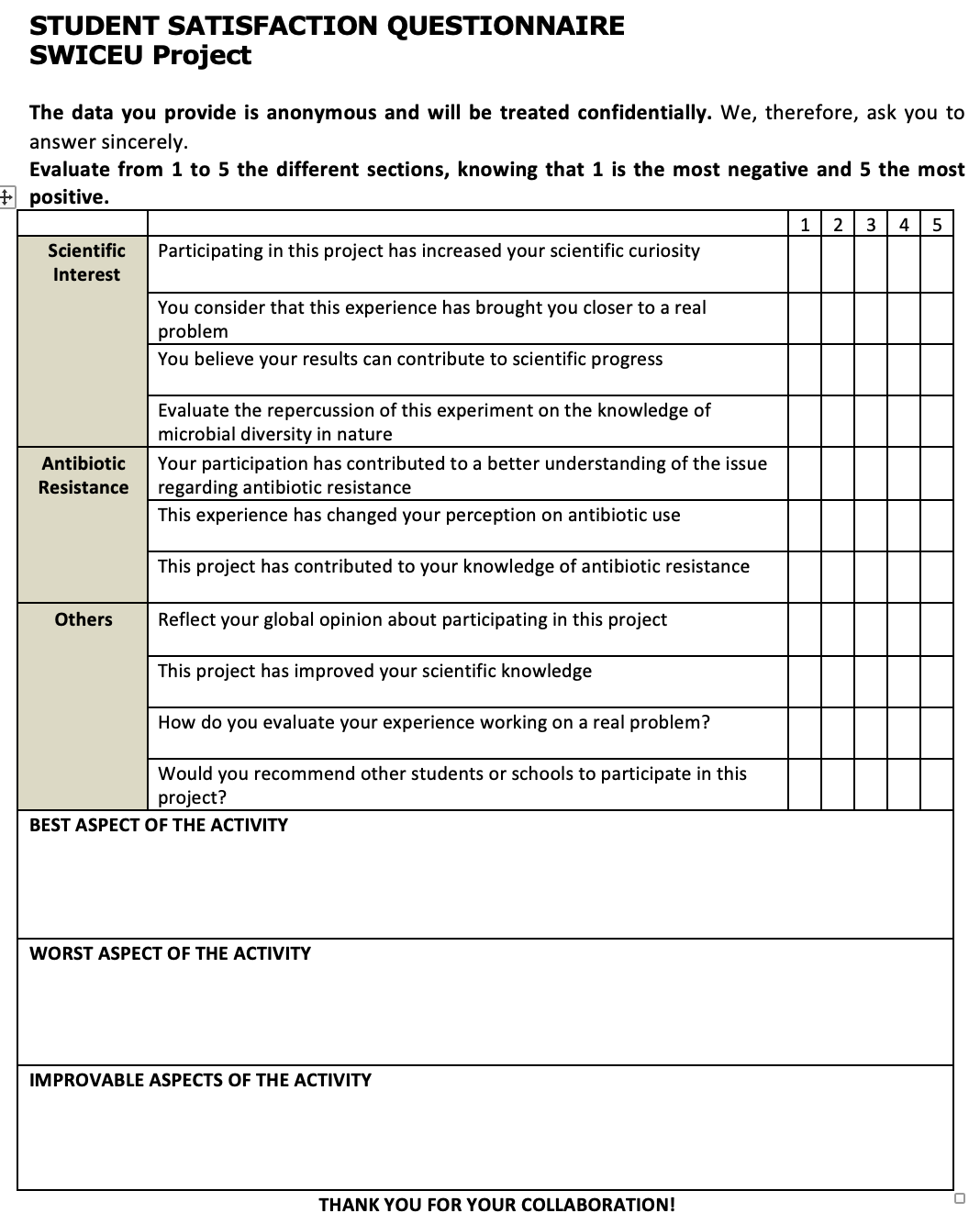


Supplementary Figure 2. Satisfaction survey carried out by pre-university students during the 5 academic years 2017-21 of the SWICEU project.


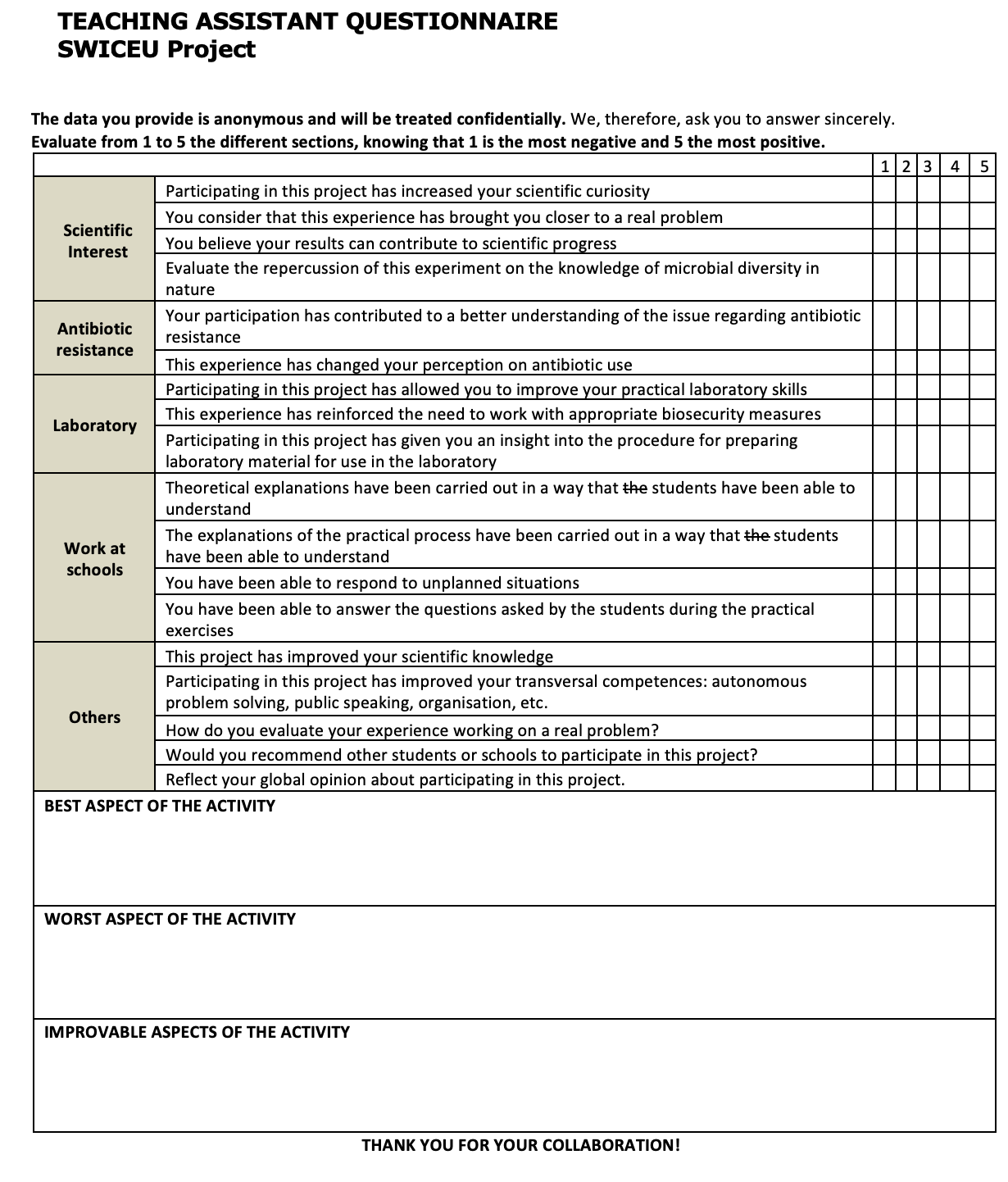


Supplementary Figure 3. Satisfaction survey conducted by university students during the 5 academic years 2017-21 of the SWICEU project.


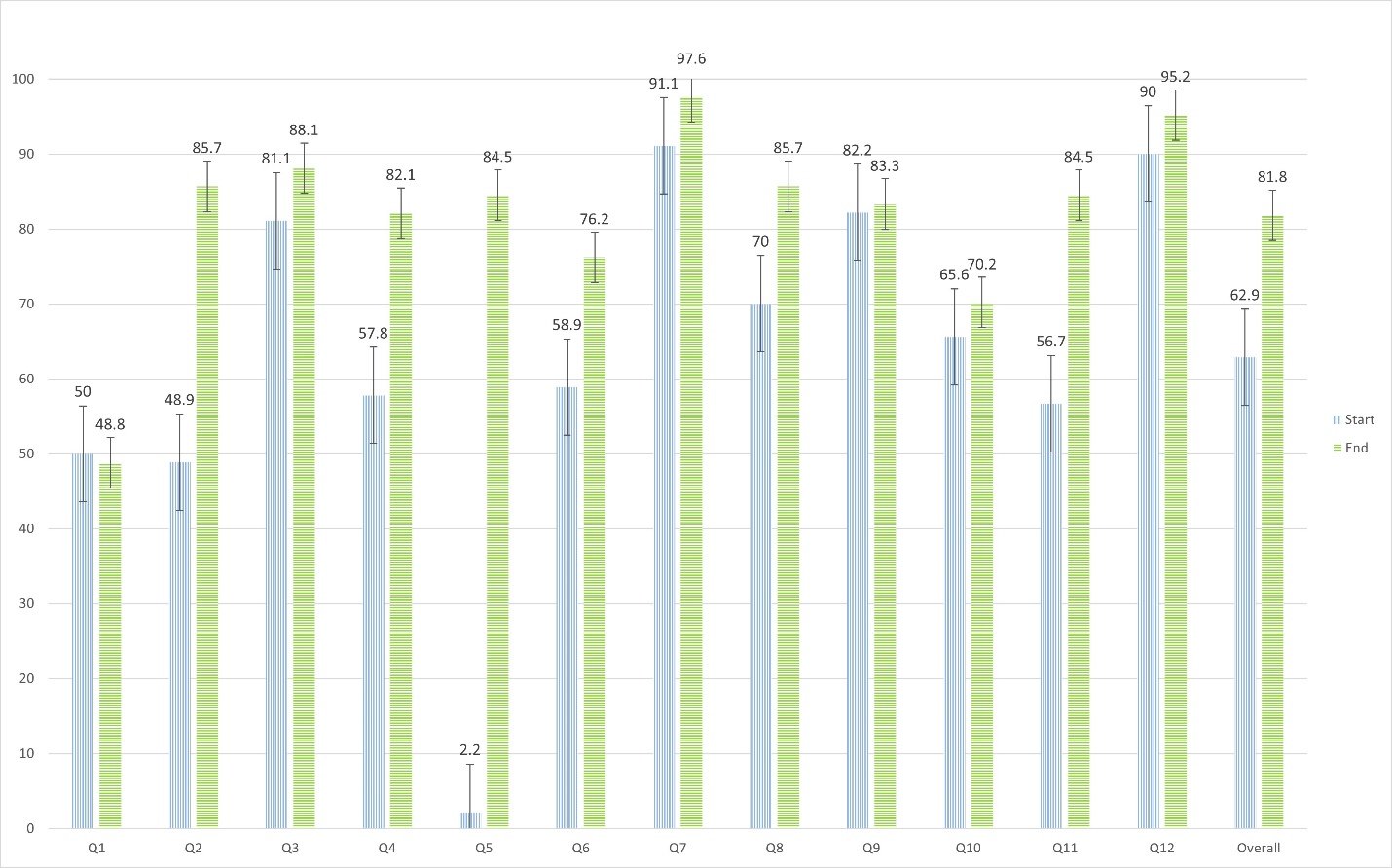


Supplementary Figure 4. Percentages of correct answers to the 12 questions of the survey carried out by pre-university students at the beginning and end of the SWICEU project during academic year 2017-2018.


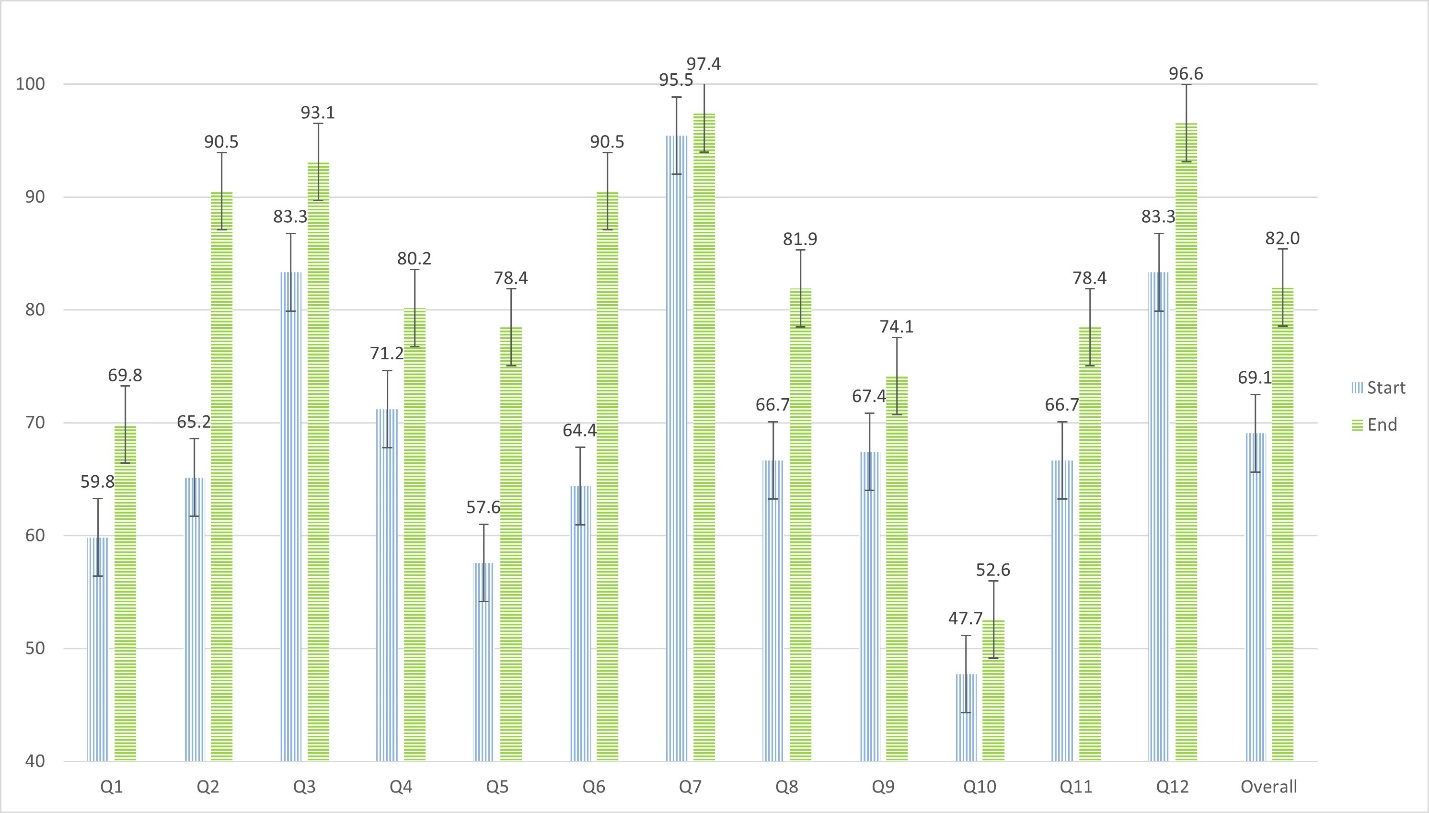


Supplementary Figure 5. Percentages of correct answers to the 12 questions of the survey carried out by pre-university students at the beginning and end of the SWICEU project during academic year 2018-2019.


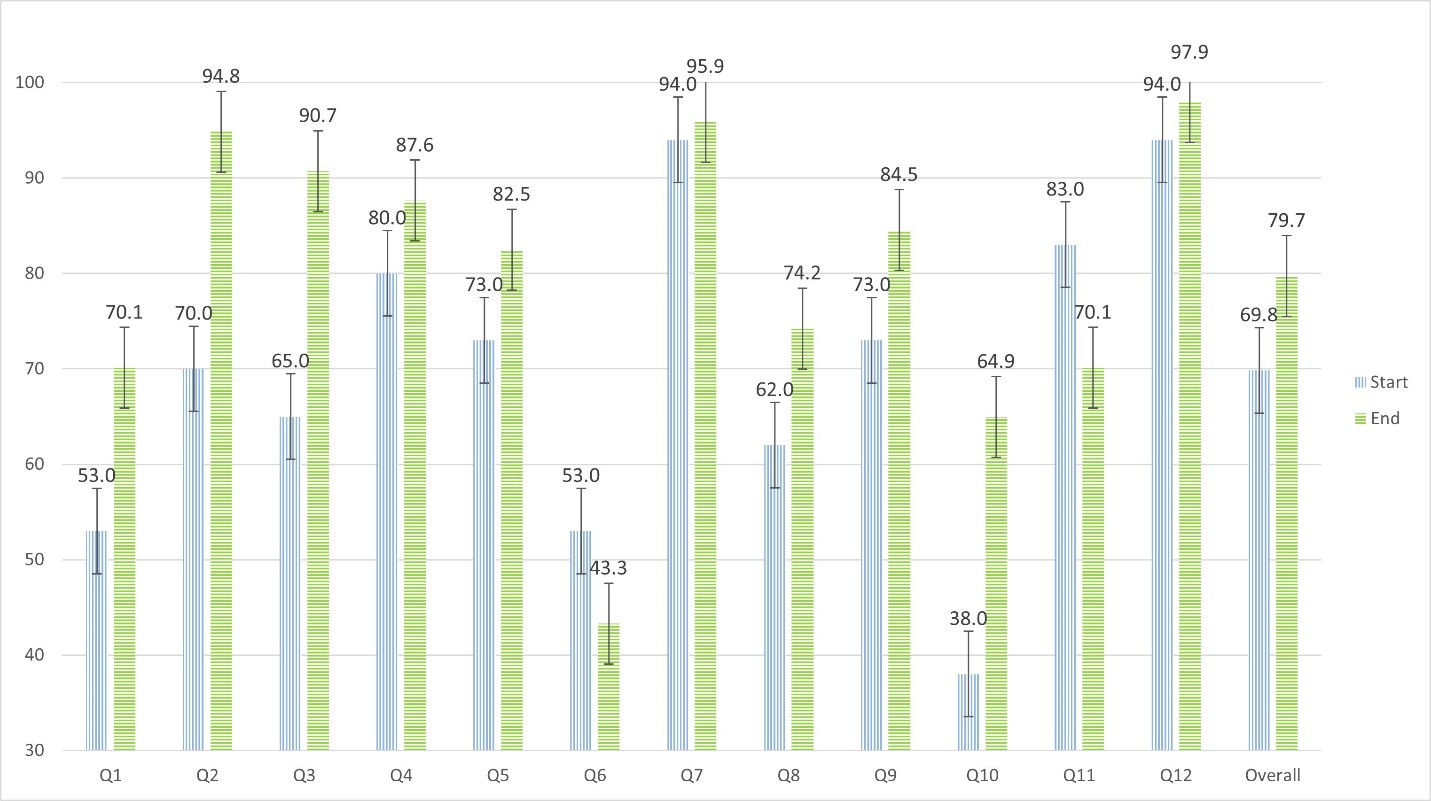


Supplementary Figure 6. Percentages of correct answers to the 12 questions of the survey carried out by pre-university students at the beginning and end of the SWICEU project during academic year 2019-2020.


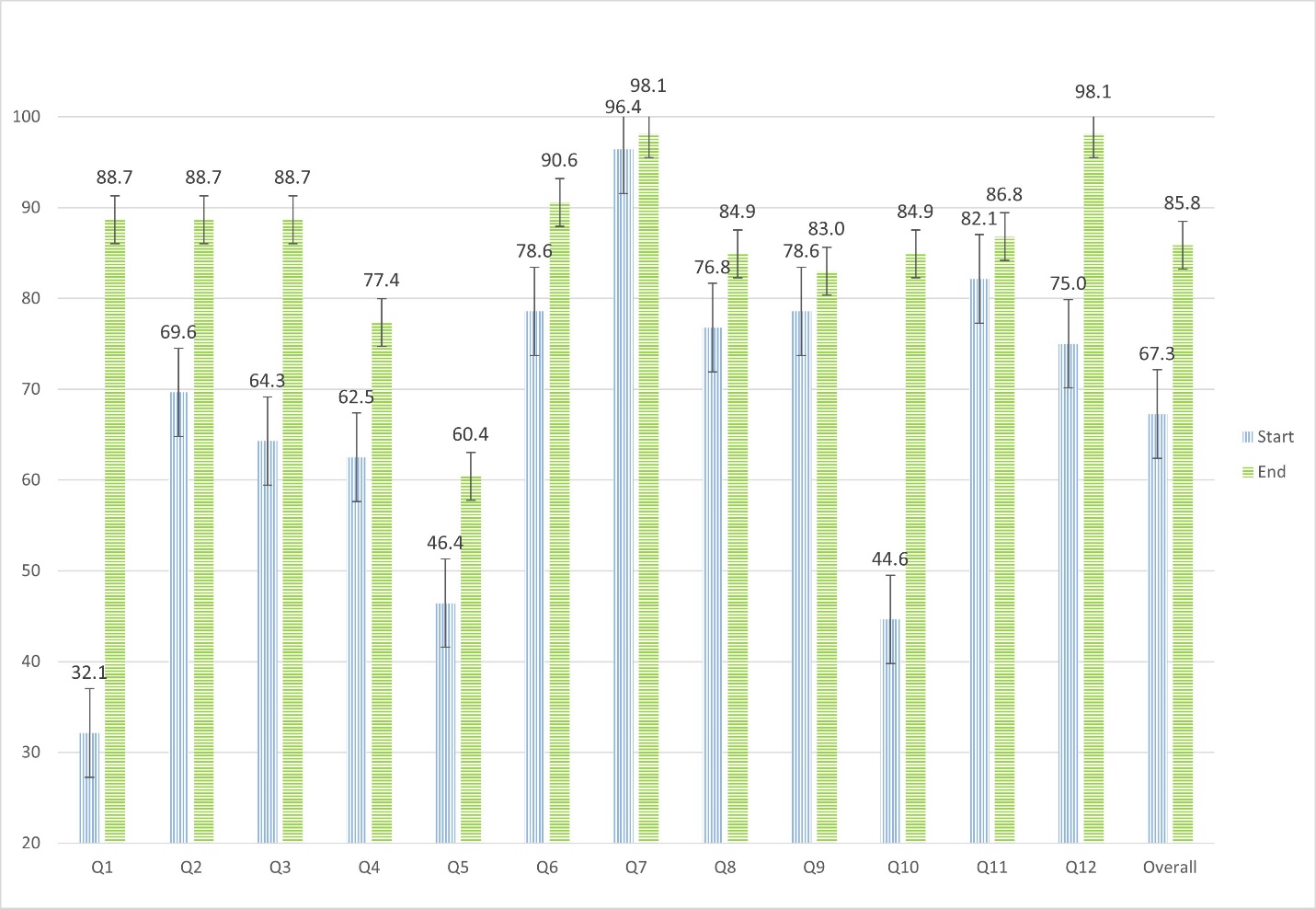


Supplementary Figure 7. Percentages of correct answers to the 12 questions of the survey carried out by pre-university students at the beginning and end of the SWICEU project during academic year 2020-2021.


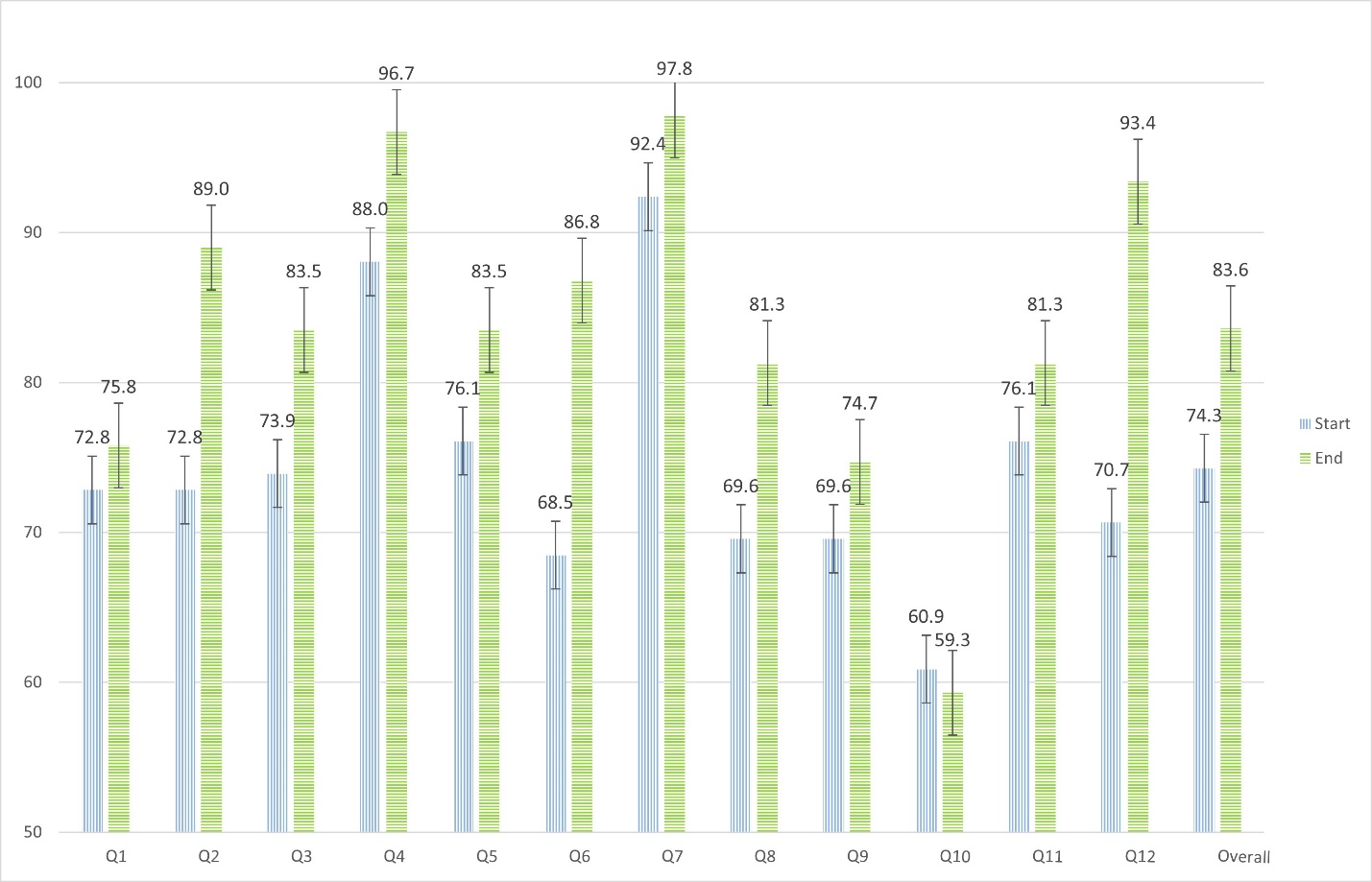


Supplementary Figure 8. Percentages of correct answers to the 12 questions of the survey carried out by pre-university students at the beginning and end of the SWICEU project during academic year 2021-2022.


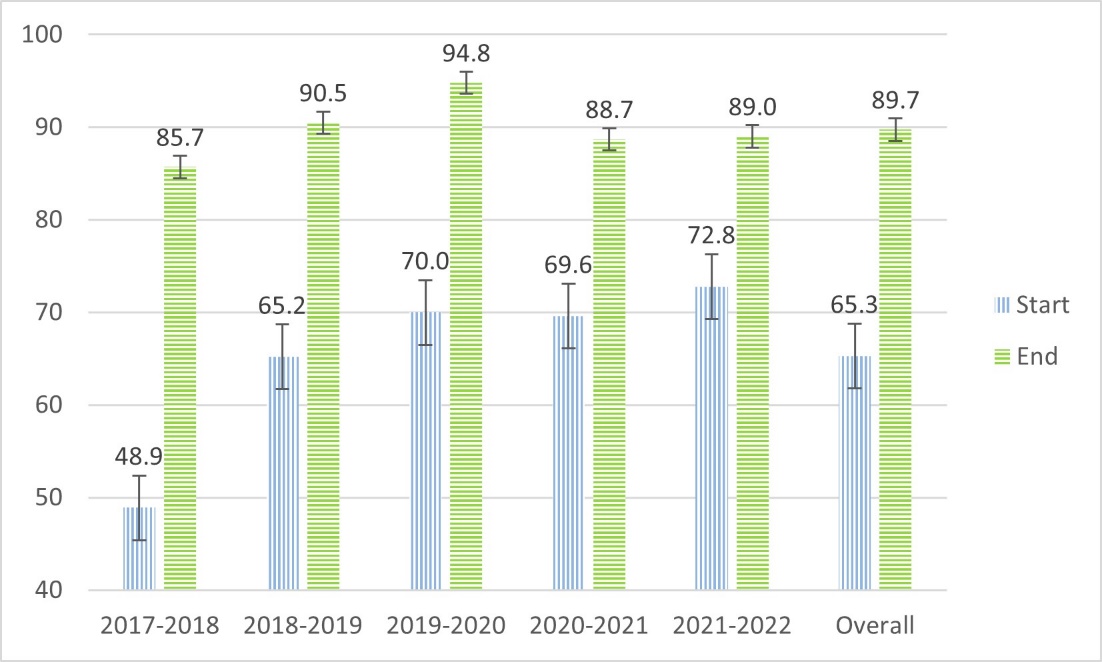


Supplementary Figure 9. Percentages of correct answers to question 2 "Is a prescription necessary to buy antibiotics?" at the beginning and end of the SWICEU project during the 2017-2021 academic years.


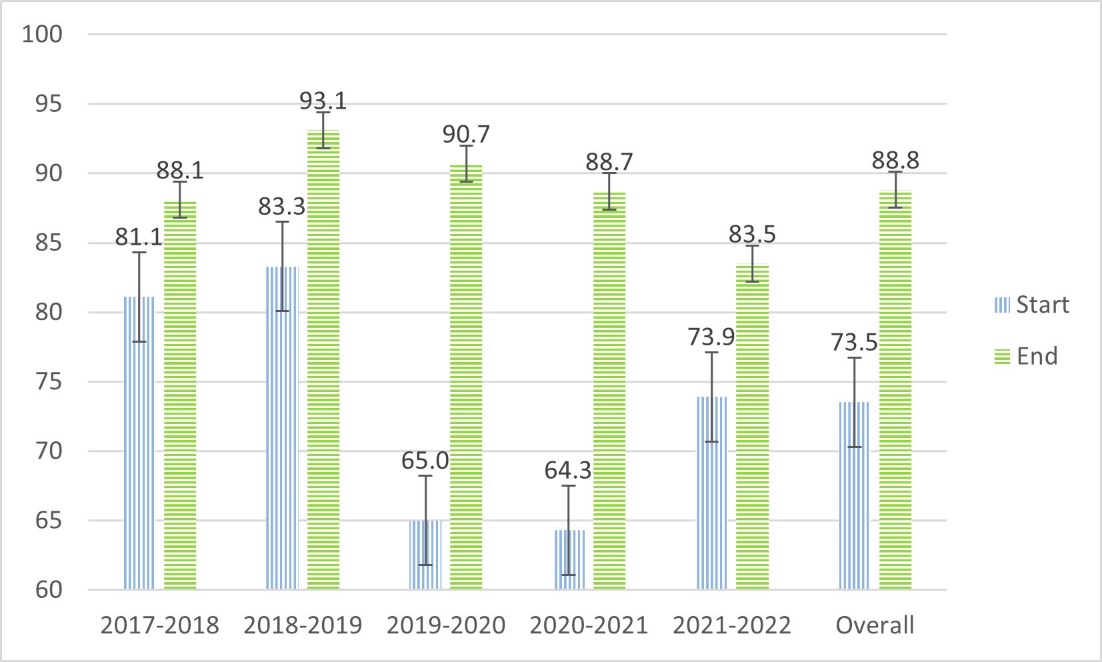


Supplementary Figure 10. Percentages of correct answers to question 3 "Which of the following drugs is an antibiotic?" at the beginning and end of the SWICEU project during the 2017-2021 academic years.


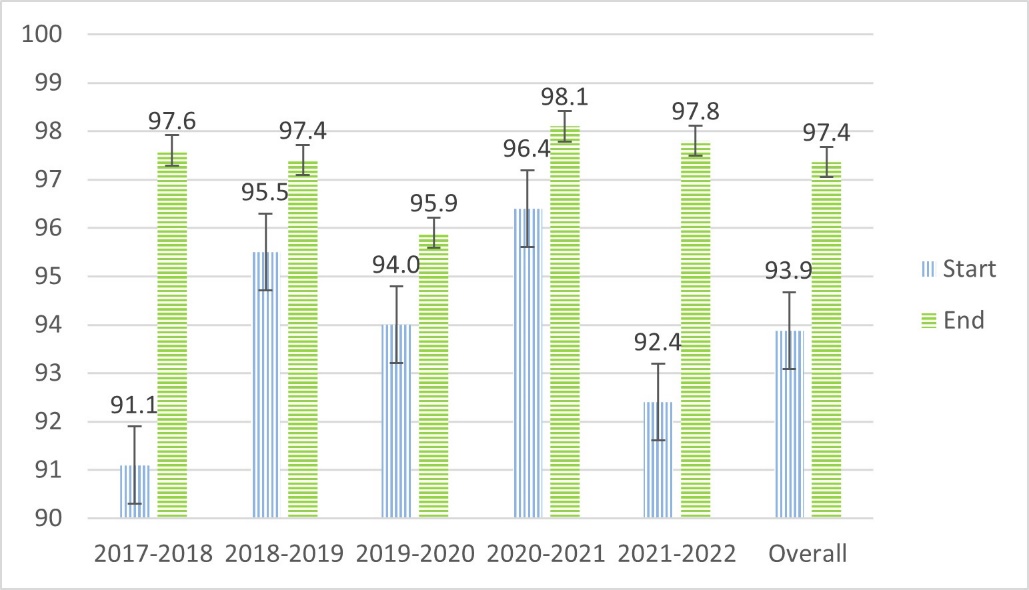


Supplementary Figure 11. Percentages of correct answers to question 7 "If we do not take antibiotics correctly:" at the beginning and end of the SWICEU project during the 2017-2021 academic years.


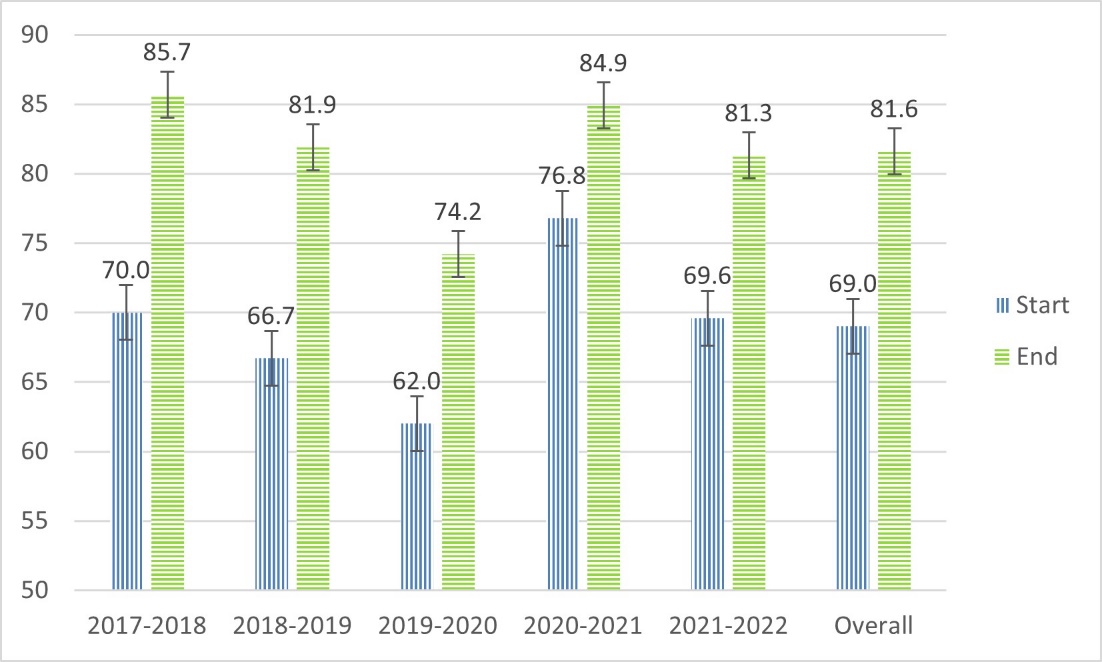


Supplementary Figure 12. Percentages of correct answers to question 8 "What is antibiotic resistance?" at the beginning and end of the SWICEU project during the 2017-2021 academic years.


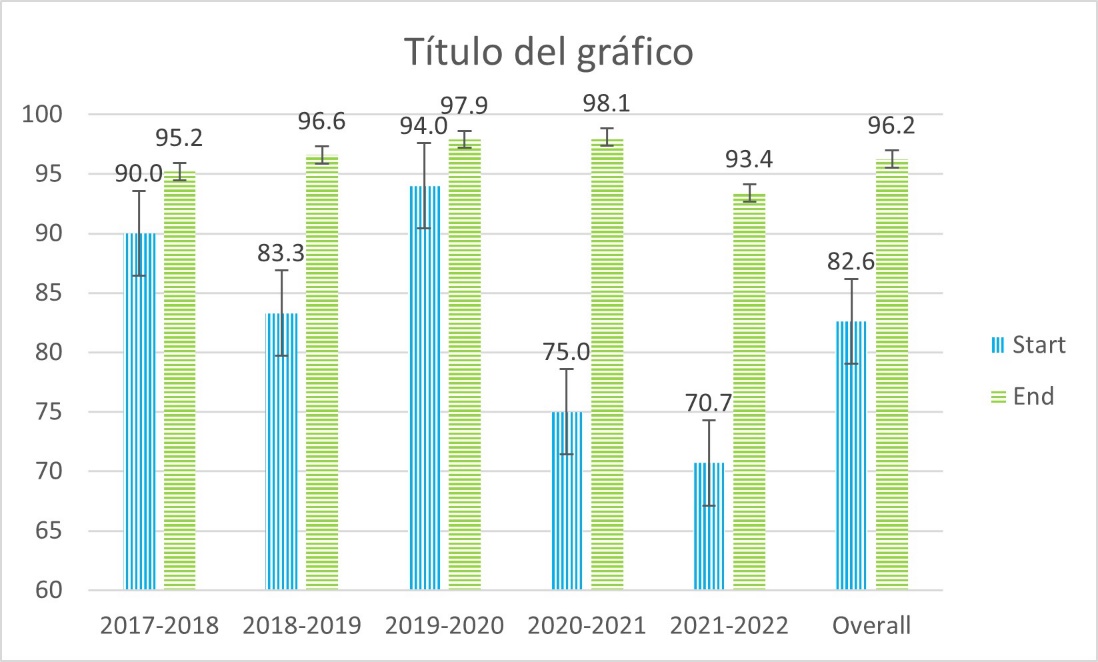


Supplementary Figure 13. Percentages of correct answers to question 12 "According to the World Health Organization (WHO), in 2050 it is estimated that the first cause of death will be:" at the beginning and end of the SWICEU project during the 2017-2021 academic years.
